# Postoperative complicated peripheral cortical cataract after ultrasound cycloplasty: a case report

**DOI:** 10.1186/s12886-020-01743-z

**Published:** 2021-01-07

**Authors:** Jihan Luo, Zhen Liu, Lin Zhao, Yi Zhou, Li Kong, Yang Sun

**Affiliations:** 1 Department of Ophthalmology, Chongqing General Hospital, University of Chinese Academy of Science, CGH, UCAS, Chongqing, China; 2 Chongqing Aier Eye Hospital, No.2, Huatang Road, Jiangbei District, Chongqing, China

**Keywords:** Ultrasound cycloplasty, Complicated peripheral cortical cataract, Pupil deformation, Case report

## Abstract

**Background:**

Ultrasound cycloplasty (UCP) is a non-invasive procedure for glaucoma treatment. Using high-intensity focused ultrasound to work on the ciliary body, the generation of aqueous humor can be reduced and the drainage of aqueous humor through the uveoscleral pathway can be enhanced. Recently, this therapy is gradually gaining clinical recognition. We report a case of a patient with glaucoma who accepted UCP in another hospital, but because of a worsening of a preexistent cataract and an insufficient IOP lowering effect, finally underwent cataract surgery in both eyes in our hospital, during the surgery we observed the unusual opacities probably due to UCP mistreatment.

**Case presentation:**

Patient was diagnosed as chronic angle closure glaucoma and catacract, accepted UCP on both eyes in another hospital 4 months ago. After the UCP therapy, the pupil was vertical ellipse, the UCP didn’t have a sufficient effect on IOP and forced us to do cataract surgery to lower IOP. During the cataract surgery, some unusual white opacities in the peripheral cortex with clear boundary were found. Inaccurate WtW measurement was the most likely cause of the injury, which resulted in the use of the small-size UCP probe and the downward movement of the UCP probe.

**Conclusion:**

UCP should not be a first line treatment in a patient with cataract and angle closure glaucoma, cataract extraction is a better choice. The appropriate case selection needs to be more strict and the preoperative indexes measurements need to be more accurate.

## Background

The treatment of glaucoma via ciliodestruction by high-intensity focused ultrasound was originally developed by Lizzi and Coleman. In the late 1980s, Sonocare manufactured an FDA-approved instrument for this treatment [1]. Ultrasound cycloplasty (UCP) is a non-invasive procedure for glaucoma treatment. Using high-intensity focused ultrasound to work on the ciliary body, the generation of aqueous humor can be reduced and the drainage of aqueous humor through the uveoscleral pathway can be enhanced. Recently, this therapy is gradually gaining clinical recognition. However, its safety and efficacy on patients have not been confirmed in a large sample size. We report a case of a patient with glaucoma who accepted UCP in another hospital, but because of a worsening of a preexistent cataract and an insufficient IOP lowering effect, finally underwent cataract surgery in both eyes in our hospital, during the surgery we observed the unusual opacities probably due to UCP mistreatment.

## Case presentation

**Fig. 1:**
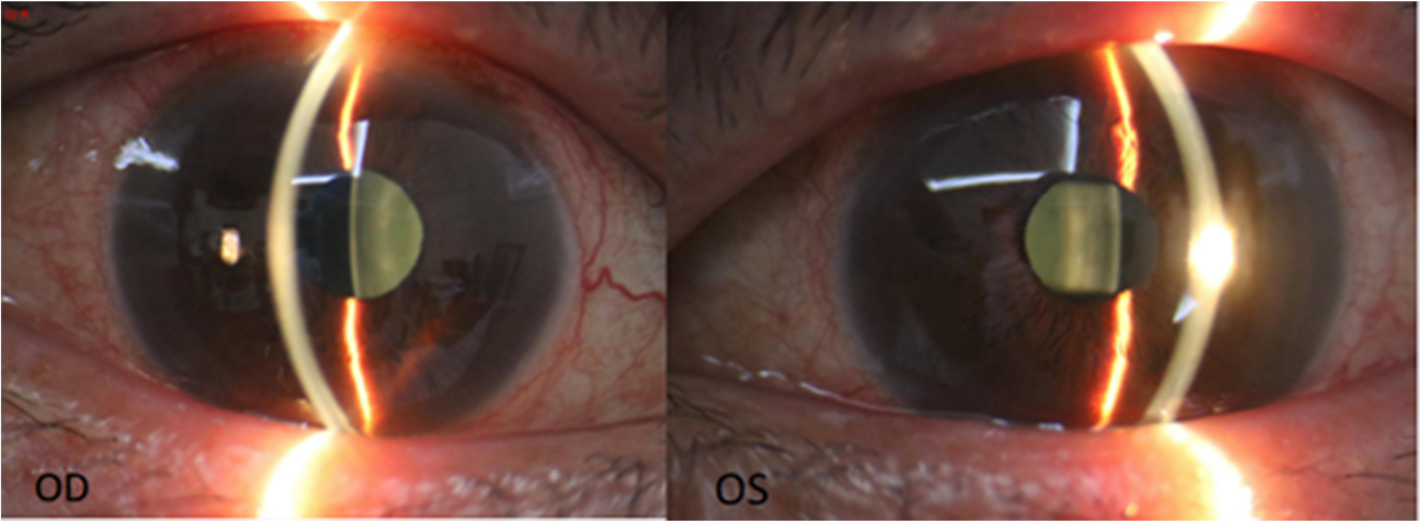
Preoperative image of UCP: Both pupils are regular circular and the anterior chamber depth is normal

**Fig. 2:**
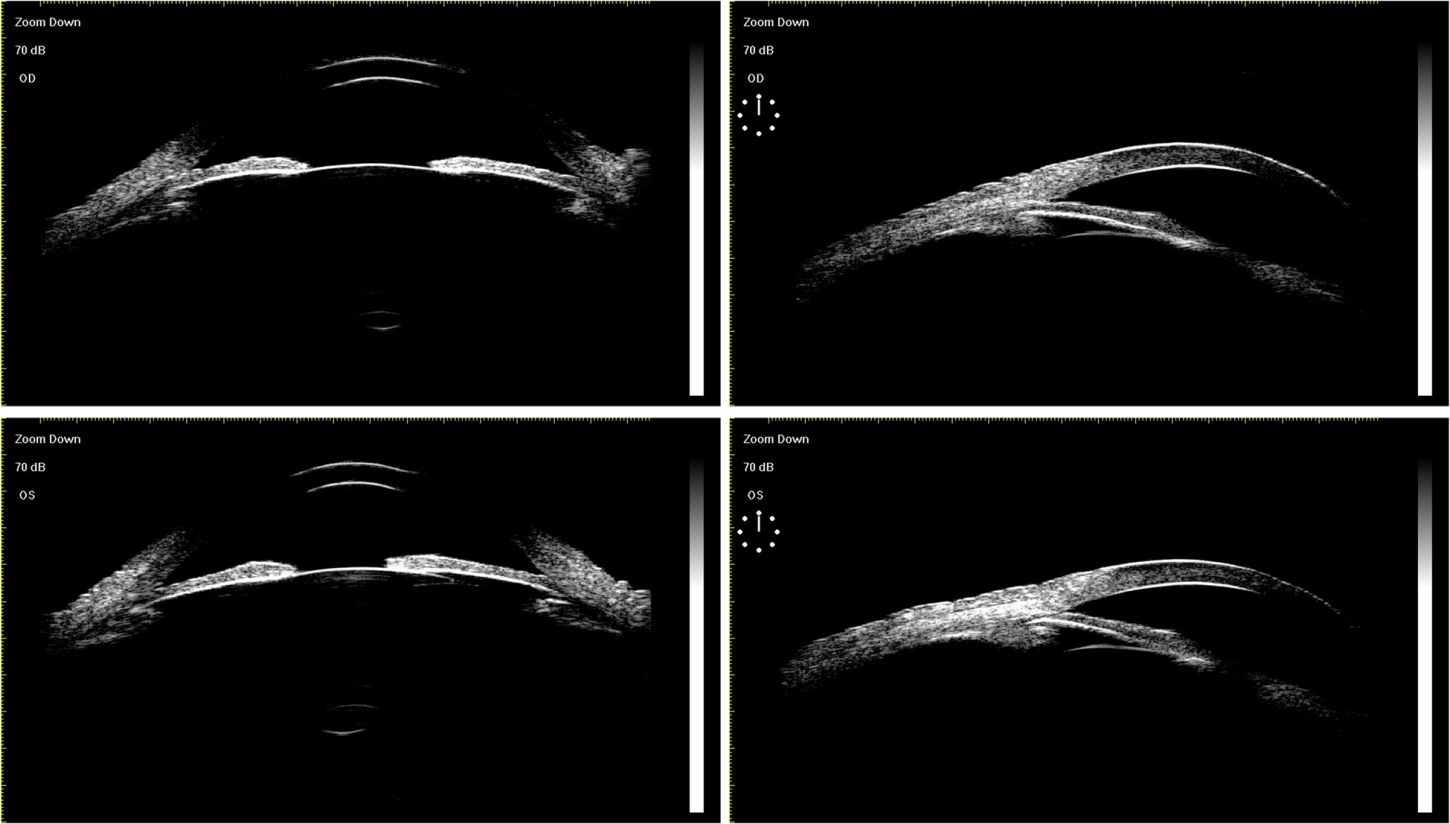
Preoperative UBM image of UCP: Both eyes have anterior chamber angle closure, the lens position is normal without deviation, and the anterior capsule is smooth without adhesion

The patient is a 63-year-old male who was diagnosed with chronic angle-closure glaucoma and accepted UCP in another hospital on both eyes 4 months ago. We asked the hospital for the preoperative data of the patient. The visual acuity was 20/160 (OD) and 20/40 (OS), and the best-corrected visual acuity (BCVA) was preserved at 20/100 (OD) with + 0.50D/− 1.50D*10 and 20/25 (OS) with + 0.75D/− 1.50D*165. The patient presented with bilateral cataract (C2N2P1 OD, C2N2P1 OS) according to Lens Opacities Classification System III (LOCS III). The optic discs of both eyes were pale with C/D = 0.9 (OD) and C/D = 0.8 (OS), respectively. The ultrasound biomicroscope (UBM) data were as follows: The iris of both eyes was swollen and the anterior chambers were narrow. In the right eye, the vertical anterior chamber depth was 2.24 mm and the corresponding sulcus-to-sulcus distance was 12.11 mm, and the horizontal anterior chamber depth was 2.28 mm with the corresponding sulcus-to-sulcus distance of 11.80 mm. In the left eye, the vertical anterior chamber depth was 2.26 mm and the corresponding sulcus-to-sulcus distance was 11.98 mm, and the horizontal anterior chamber depth was 2.24 mm with the corresponding sulcus-to-sulcus distance of 11.90 mm. Visual field examination showed that the right eye had a tubular visual field and the left eye had a peripheral visual field defect. Under the situation of using three kinds of anti-glaucoma drugs (methazolamide, carteolol hydrochloride, and brinzolamide), the baseline intraocular pressure (IOP) was 30 mmHg (OD) and 31 mmHg (OS), respectively. The preoperative anterior segment image (Fig. 1) showed that pupils of both eyes were regular circular and the depth of the anterior chamber was normal. The preoperative UBM image (Fig. 2) presented that the anterior chamber angle of both eyes was closed, the lens position was normal without deviation, and the anterior capsule was smooth without adhesion.

After evaluation, the hospital decided that the patient was suitable for UCP. We, on the other hand, have reservations about the decision and will elaborate on our reasons in the discussion section. The parameters of the operation were as below: OD: white-to-white (WtW, measured by calipers, IOL-master and corneal topography) distance = 11.4 mm, axial length (AL) = 23.21 mm; OS: WtW distance = 11.4 mm, AL =23.03 mm. A high-intensity focused ultrasound (HIFU) equipment (EYE TECH CARE, sound glaucoma care, Version 3.20) was used for the UCP treatment. The parameters were as follows: Vacuum setpoint 225 mmHg; Duration 08.0 S; Pause 20.0 S; Probe Size 12 mm; Model T4T. SN = 18/172/018 / Unit = 15/007 (OD). SN = 18/172/013 / Unit = 15/007 (OS). UCP prepared for 10 sectors, but only 8 sectors treated (5 superior area/3 inferior area) on both eyes.

**Fig. 3:**
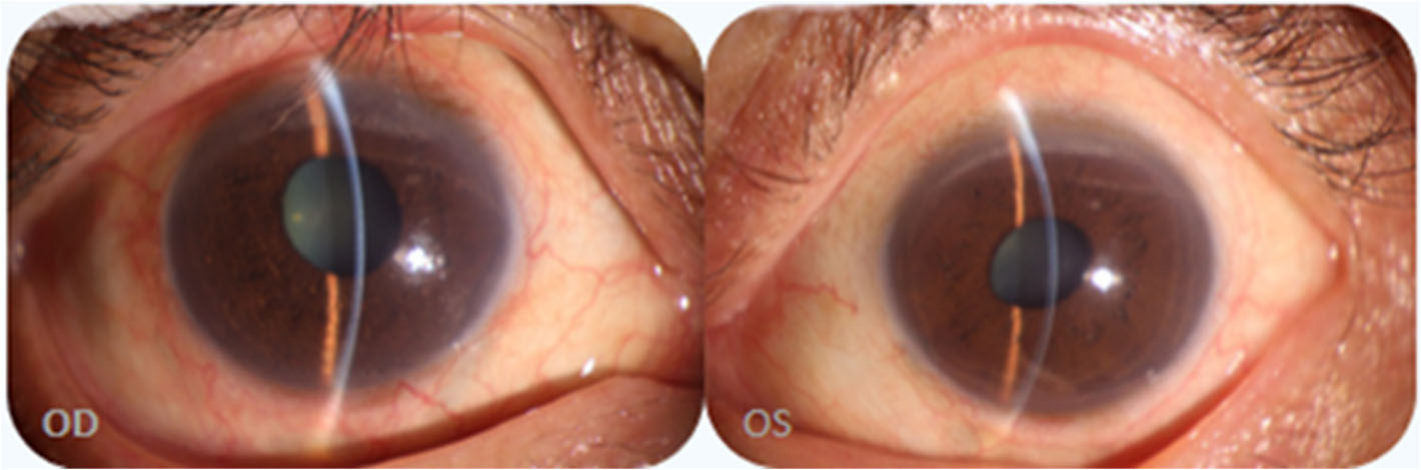
Postoperative image of UCP: The diameters of two pupils were 3.0 mm*5.0 mm (OD) and 3.0 mm*4.0 mm (OS), respectively. Local iris atrophy on the subnasal of the left eye was observed. The central anterior chamber depth is about 2.5 corneal thickness (CT). The left eye had a peripheral anterior chamber depth of 1/4 CT while that of the right eye varied greatly, which indicates an angle adhesion

**Fig. 4:**
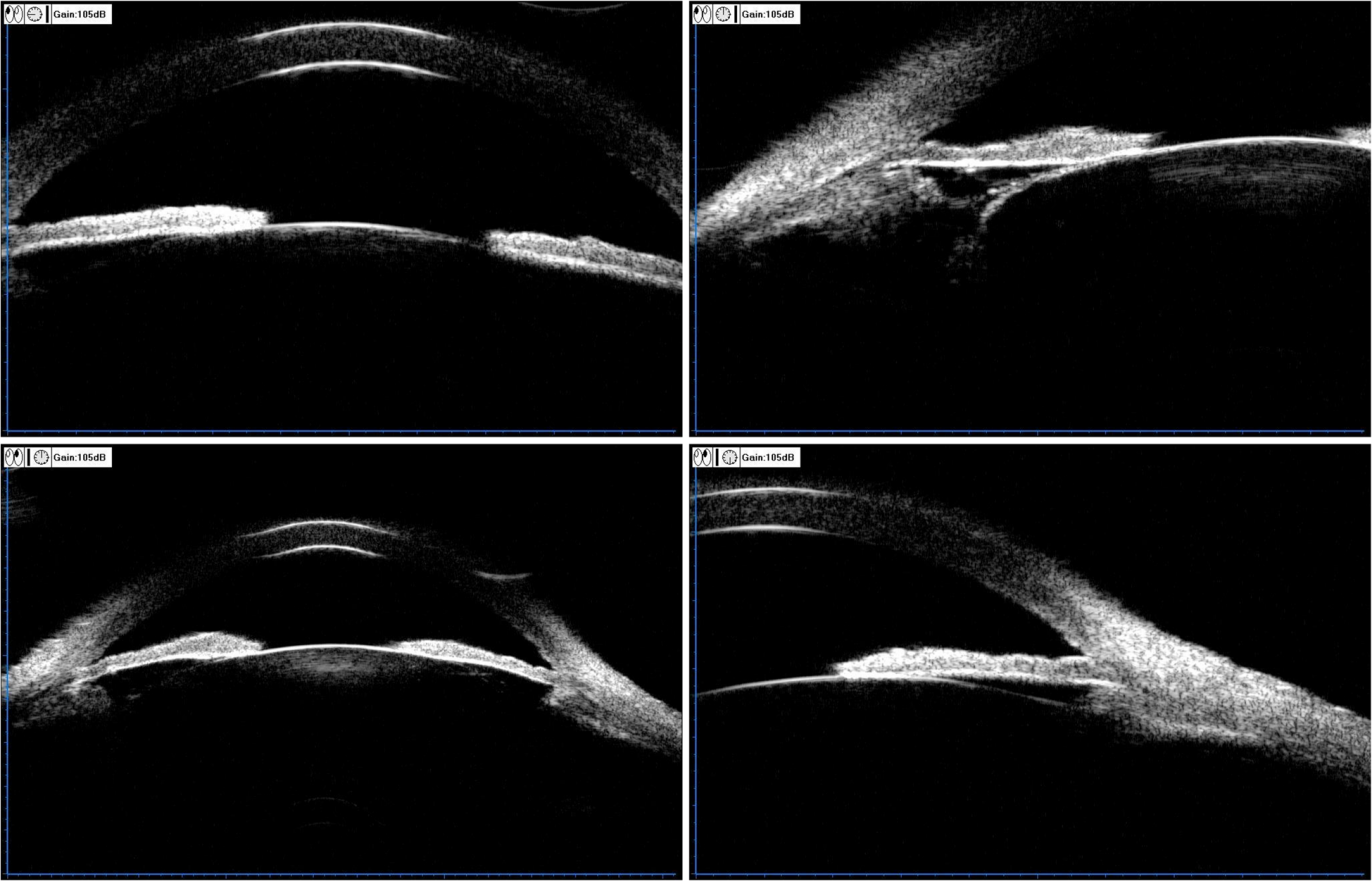
Postoperative UBM image of UCP: The central anterior chamber depth is about 2.32 mm (OD) and 2.25 mm (OS), respectively. The anterior chamber angle was closed in all directions and the ciliary body displaced forward to the root of the iris in both eyes. The shape of the superior suspensory ligament is irregular in the left eye. Both lenses show enhanced echo. There exists an adhesion in the anterior capsule periphery in both lenses while the lens position of the right eye deviates

Three months after the UCP treatment, under the situation of using three kinds of anti-glaucoma drugs (methazolamide, carteolol hydrochloride, and brinzolamide), the IOP value of the patient fluctuated in the range of 22–30 mmHg and the visual acuity decreased to 20/250 (OD) and 20/80 (OS). It was found that the BCVA was preserved at 20/40 (OD) with − 1.25 DC*35 and 20/32 (OS) with + 2.50DS/− 3.00 DC*155. After the UCP treatment, the image of the anterior segment (Fig. 3) exhibited that both pupils were vertical elliptical, of which the diameters were 3.0 mm*5.0 mm (OD) and 3.0 mm*4.0 mm (OS), respectively. Also, local iris atrophy on the subnasal of the left eye was observed. The central anterior chamber depth was about 2.5 corneal thickness (CT). The left eye had a peripheral anterior chamber depth of about 1/4 CT while the peripheral anterior chamber depth of the right eye varied greatly, which indicates an angle adhesion. The patient presented with bilateral cataract (C2N3P1 OD, C2N2P1 OS) according to LOCS III. The UBM image (Fig. 4) showed that both pupils were deformed, in which the left eye had an irregular superior suspensory ligament and the lens of the right eye deviated. The central anterior chamber depth was about 2.32 mm (OD) and 2.25 mm (OS), respectively. As shown in the image, the anterior chamber angle was closed in all directions while the ciliary body displaced forward to the root of the iris in both eyes. Both lenses showed enhanced echo. It is seen that there exists adhesion in the anterior capsule periphery of both lenses and the lens position of the right eye deviates.

**Fig. 5:**
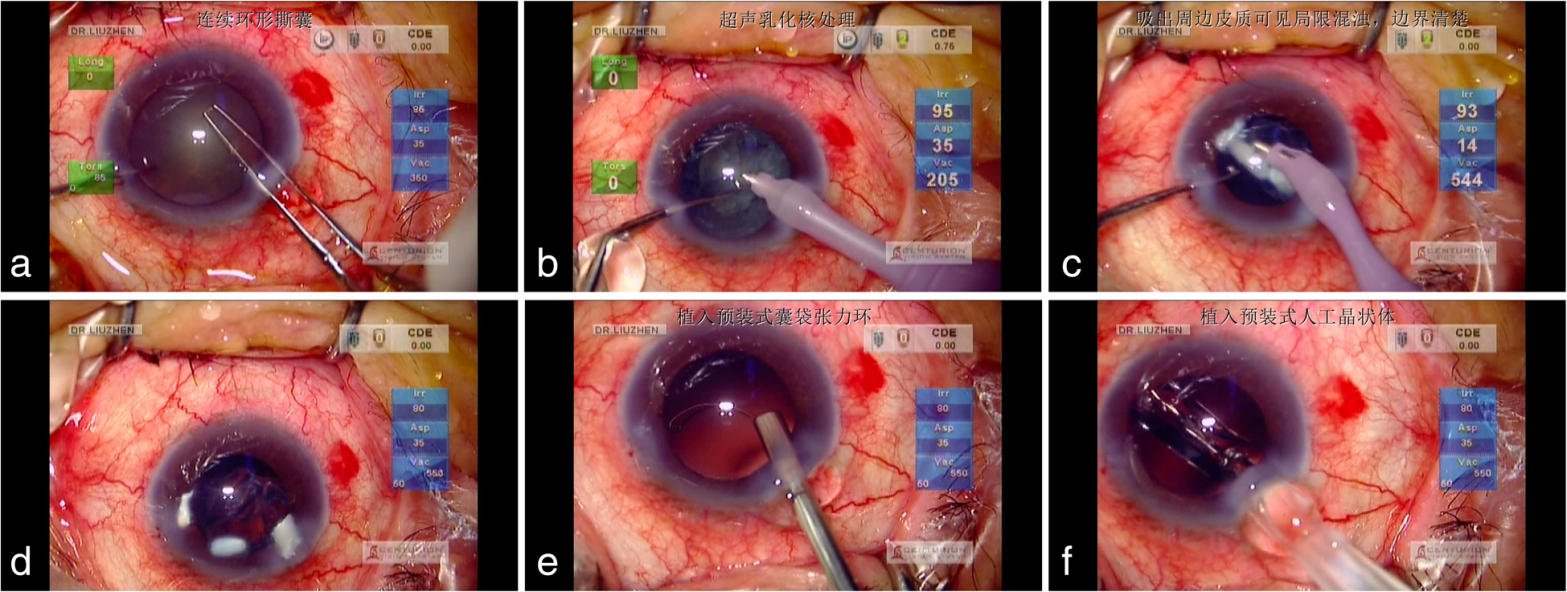
Process of phacoemulsification: a Continuous curvilinear capsulorhexis; b Phacoemulsification; c In the process of phacoemulsification, several continuous localized white opacities with clear boundaries are found in the peripheral cortex; d The single image of the white opacities in the peripheral cortex; e A capsule tension ring is planted into the eye; f Intraocular lens implantation

Thus, we decided to perform cataract surgery on the right eye of the patient. Considering the lens position deviation and irregular suspensory ligament, we planned to implant a capsule tension ring into the eye to maintain the stability of the capsular bag. During the process of phacoemulsification, as Fig. 5 showed, several continuous localized white opacities in the peripheral cortex with clear boundaries were observed, which could not be found before the operation even if after mydriasis. Because the opacities were in the equator part of the lens, it could only be found when they were dragged out by I/A piece. At last, an intraocular lens was implanted into the eye. The 5-day postoperative visual acuity was 20/50, and the IOP value was 22 mmHg. After taking two anti-glaucoma drugs (carteolol hydrochloride and brinzolamide), the IOP value dropped to 8 mmHg 3 days later.

**Fig. 6:**
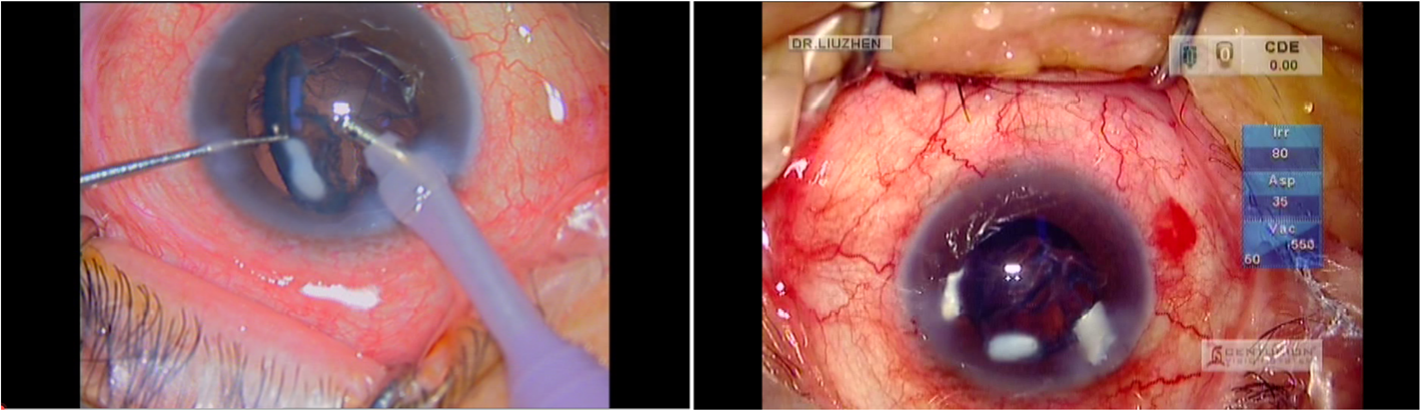
Peripheral cortical cataract: Opacities were all located in the equator part of both lenses, which could not be seen by the shelter of pupils until they were dragged out. The opacities in the right eye were four separated pieces while the opacity of the left eye was a whole piece. The common thing is that they are all white calcified plaques

Five days after the surgery of the right eye, cataract surgery was taken on the left eye. It was also found in the process of I/A that there was a large white opacity formed by the fusion of several pieces of calcification in the equator part of the lens. As Fig. 6 showed, although the opacities in the right eye were four separated pieces while the opacity in the left eye was a whole piece, they were all white calcified plaques.

## Discussion

Using a small computer-controlled eye probe to focus the high-intensity ultrasound energy on the ciliary body, UCP facilitates epithelium to generate aqueous humor in a non-incision, quantifiable, and accurate way. In general, the thermal effect of ultrasound is theoretically controllable within the submillimeter level. In this case, however, the continuous localized white opacities are so regular with markedly clear boundaries, which is neither any kind of natural cataract we know nor reported in any literature before. So it leads to a question that whether the ultrasound thermal injury causes the cataract. The location of those opacities is in the equator of the lens, where is just below the ciliary body. The shape of opacities also matches the effected area of ultrasound thermal damage according to some early research [2].

After the UCP treatment and cataract surgery, the IOP value of the patient still cannot reach an ideal level until the intervention of anti-glaucoma drugs. Therefore, we need to decide whether additional glaucoma surgery is necessary for the next step [3]. The pupil ovalization and accommodation loss and iatrogenic corneal astigmatism after UCP treatment also interfere with the visual acuity recovery of the patient in this case, which also has been reported by some other researches [4–6].

**Fig. 7:**
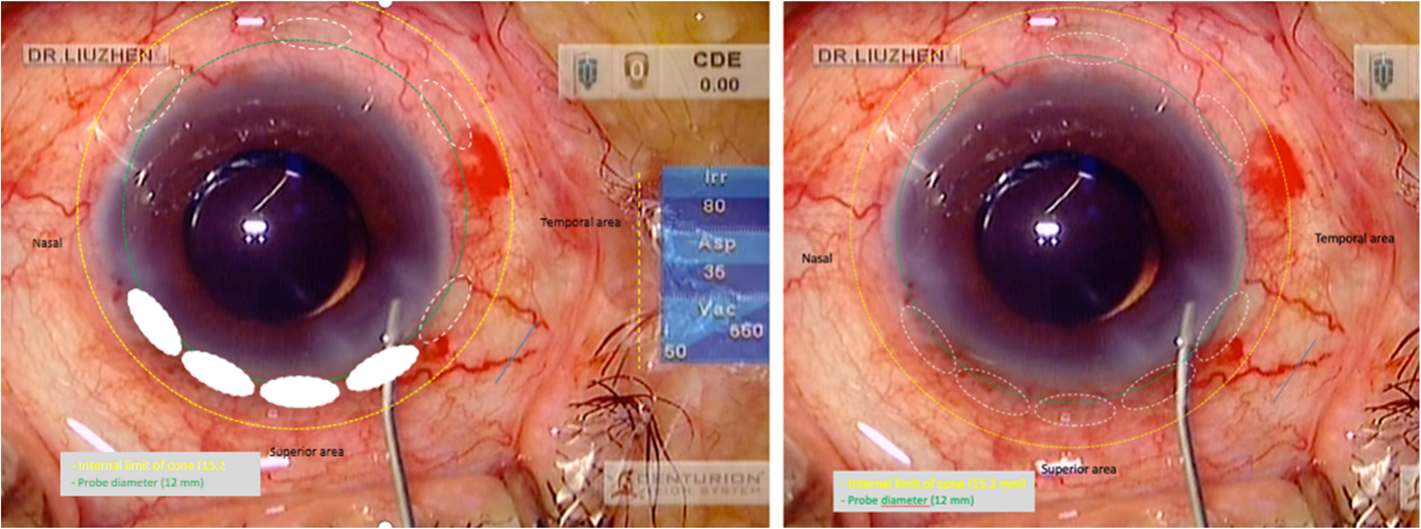
Nonoptimal centering and optimal centering simulation (OD): The left figure illustrated a displacement of the probe and 4 lesions into the lens based on the 4 scleral marks (dotted line). The right figure showed the optimal centering simulation

After careful retrospective analysis of the UCP procedure of this case, we reckoned that the inaccurate WtW measurement was the most likely cause of the injury, which might result in the use of the small-size UCP probe and the downward movement of the UCP probe during the surgery. Several papers [7–9] have proved that the WtW distance does not necessarily correspond to the sulcus-to-sulcus distance using UMB and the former distance is an inaccurate indicator predicting the position of the ciliary processes. Figure 7 was a simulation of the nonoptimal centering and optimal centering (OD). The left figure illustrated a displacement of the probe and 4 lesions (full line) into the lens based on the 4 scleral marks (dotted line). The right figure showed the optimal centering simulation. Just because of the downward movement of the probe, the upper equatorial lens was injured by accident, forming a calcification with clear boundaries caused by ultrasonic thermal damage.

We are not sure whether such complicated peripheral cortical cataracts are frequent but lack of coverage due to concealment, or just an individual case of improper manipulation and improper selection of treatment patients. At least, however, in this case, we believe the best operation for this patient is cataract surgery rather than UCP. Cataract extraction is a well-accepted indication for angle-closure glaucoma, whereas UCP is a second-line treatment. In other words, if the patient is diagnosed with angle-closure glaucoma with cataract, we would choose cataract extraction as the first therapy.

According to the existing literature [10], indications for UCP include 1. Various refractory glaucomas such as primary open-angle glaucoma, primary angle-closure glaucoma, secondary glaucoma (for instance, neovascular glaucoma, secondary glaucoma after cataract surgery, glaucoma after corneal transplantation, etc.), and refractory glaucoma with uncontrolled intraocular pressure after multiple glaucoma surgeries; 2. No vision and no possibility of vision recovery; 3. No surgery opportunity and no surgery value; 4. Severe pain; 5. Poor general conditions that unable to tolerate surgery.

However, based on the complications this patient suffered, we believe that applying UCP treatment needs more caution and discretion. We should pay more attention to whether the indications of UCP in the clinical application are too wide, or whether some other problems have been not observed or reported yet.

## Conclusion

In this case, the UCP didn’t have a sufficient effect on IOP and forced us to do cataract surgery to lower IOP. This lack of effect was due to the misplacement of the probe that caused the lens opacities. UCP should not be a first line treatment in a patient with cataract and angle closure glaucoma, cataract extraction is a better choice. To maximize the efficacy of UCP and minimize the patients’ loss, the appropriate case selection needs to be more strict and the preoperative indexes measurements need to be more accurate.

## Data Availability

The datasets generated and/or analysed during the current study are not publicly available due to an invasion of individual privacy but are available from the corresponding author on reasonable request.

## Abbreviations

UCP: Ultrasound cycloplasty
IOP: Intraocular pressure
BCVA: Best-corrected visual acuity
UBM: Ultrasound biomicroscope
CT: Corneal thickness
LOCS III: Lens Opacities Classification System III
